# *Adu*CPI2 alleviates MSU-induced acute gouty arthritis in mice by inhibiting cathepsin S and the C5a-C5aR1 axis

**DOI:** 10.3389/fphar.2025.1604329

**Published:** 2025-06-13

**Authors:** Sijing Liu, Yongli Situ, Li Deng, Meng Liang, Haoyuan He, Zheng Shao, Lifei Peng

**Affiliations:** ^1^ Department of Orthopaedic Center, Maoming Hospital of Guangzhou University of Chinese Medicine, Maoming, China; ^2^ Department of Orthopaedic Center, The Second Hospital Affliated of Guangdong Medical University, Zhanjiang, China; ^3^ Department of Parasitology, Guangdong Medical University, Dongguan, China; ^4^ Institute of Pathogenic Biology and Immunology, Guangdong Medical University, Dongguan, China; ^5^ Department of Parasitology, Guangdong Medical University, Zhanjiang, Guangdong, China

**Keywords:** cysteine protease inhibitor, C5a-C5aR1 axis, cathepsin S, gouty arthritis, mice

## Abstract

**Background:**

Gout, a prevalent and severe form of arthritis precipitated by hyperuricemia, has been the subject of extensive research. However, the intricate underlying mechanisms governing gout-related inflammation are still only partially elucidated. *Ancylostoma duodenale* cysteine protease inhibitor 2 (*Adu*CPI2, abbreviated as CPI2, GenBank No. JQ762417) is a protein isolated from *A. duodenale* by our research group in the early stage. CPI2 can effectively inhibit the activity of cathepsin S and reduce the level of C5a. In this study, we aimed to investigate the effect of CPI2 on acute gouty arthritis stimulated by monosodium urate (MSU) crystals.

**Methods:**

The CPI2 fusion protein was induced for expression in *Escherichia coli* with isopropyl β - D - 1 - thiogalactopyranoside (IPTG). The recombinant CPI2 fusion protein was purified via Ni-NTA affinity chromatography and SP Bio-sep FF ion exchange chromatography. The fusion tag was cleaved by SUMO protease to yield the purified CPI2 protein. Sodium dodecyl sulfate-polyacrylamide gel electrophoresis (SDS-PAGE) was employed to analyze the protein expression and purification status. An enzyme activity inhibition assay was conducted to detect the inhibitory effect of CPI2 on cathepsin S. In an attempt to mimic human gouty arthritis, suspensions of monosodium urate (MSU) crystals were injected into the right foot pads of C57BL/6 wild-type (WT) mice and C5aR1^−/−^ mice. After the injection of MSU crystals, the mice were intravenously administered CPI2 at doses of 0.5, 1, and 2 mg/kg body weight. The effects of CPI2 on mice with MSU-induced gouty arthritis were evaluated by measuring the degree of paw swelling, observing the histopathological changes in paw tissues using hematoxylin and eosin (HE) staining, detecting the levels of inflammatory cytokines, C5a, and cathepsin S by enzyme-linked immunosorbent assay (ELISA), determining the activities of glutathione peroxidase (GSH-Px) and superoxide dismutase (SOD) as well as the level of malondialdehyde (MDA) by chemiluminescence assay, and observing the expression level of C5aR1 protein in paw tissues by immunohistochemistry.

**Results:**

After culturing in *E. coli*, induced expression, and isolation-purification, CPI2 with a purity of over 98% was finally obtained. CPI2 ameliorated the inflammation of MSU-induced gouty arthritis in a dose-dependent manner. It reduced joint swelling, decreased the infiltration of inflammatory cells, lowered the levels of pro-inflammatory cytokines (IL-1β, IL-6, and TNF-α), increased the level of the anti-inflammatory cytokine IL-10, enhanced the activities of SOD and GSH-Px, while reducing the content of MDA, decreasing the levels of C5a and cathepsin S, and down-regulating the expression level of C5aR1 protein. The knockout of the C5aR1 gene was beneficial for inhibiting the inflammation of MSU-induced gouty arthritis.

**Conclusion:**

CPI2 alleviates MSU-induced acute gouty arthritis in mice through the inhibition of cathepsin S and the C5a - C5aR1 axis. CPI2 may represent a potential candidate for the treatment of gouty arthritis.

## 1 Introduction

Gouty arthritis ranks among the most prevalent forms of inflammatory arthritis globally (Dalbeth et al., 2019; Topiwala et al., 2023). Its etiology is ascribed to the aberrant elevation of blood uric acid levels and the abnormal deposition of monosodium urate (MSU) crystals within and around joints. These factors act in concert, precipitating a cascade of detrimental effects (Dalbeth et al., 2021). Clinical manifestations in gout patients typically include joint pain, erythema, and fever (Tatlock et al., 2017). Despite its high clinical incidence, the underlying mechanisms of the associated inflammatory responses remain incompletely understood. Currently, while gout cannot be completely eradicated, it can be effectively managed through rational drug therapy and efficacious self - management strategies. In contemporary clinical practice, the treatment of gout predominantly relies on medications such as glucocorticoids, non-steroidal anti-inflammatory drugs, and colchicine. These drugs ameliorate gout pain principally by suppressing the inflammatory response (Neogi, 2011; Cronstein and Sunkureddi, 2013). However, it is essential to note that long - term or excessive use of these drugs frequently gives rise to a series of severe adverse effects, such as gastrointestinal toxicity, nephrotoxicity, and gastrointestinal bleeding. Moreover, there are inherent limitations in their therapeutic efficacy. These adverse effects not only pose an additional burden on patients’ health but also substantially diminish the patients’ acceptance of these traditional therapeutic agents (Terkeltaub, 2010; Qaseem et al., 2017; Schlesinger et al., 2011; Xu et al., 2022). Given the multitude of issues associated with current gout treatment drugs, there is an urgent imperative to thoroughly explore novel anti-inflammatory mechanisms for gout. Consequently, it is necessary to identify new drug targets and develop safer and more effective therapeutic agents, thereby offering improved treatment options for gout patients and enhancing their quality of life.

The complement system, a fundamental component of the innate immune response, plays a pivotal role in host defense and inflammatory processes (Gil et al., 2022). In recent years, the role of C5a and its receptors in the pathogenesis of inflammatory diseases has emerged as a research area attracting substantial attention (Guo and Ward, 2005). Dysregulation of the complement system, such as excessive or persistent C5a-mediated inflammatory responses or the formation of the membrane attack complex, can culminate in tissue damage and disease progression (Schanzenbacher et al., 2023). The complement system is implicated in the development of gouty arthritis. Activated complement components have been detected in the synovial fluid of gout patients during acute attacks (Doherty et al., 1988; Brodeur et al., 1991). One of the mechanisms by which MSU crystals instigate inflammation is through activation of the complement system (Giclas et al., 1979). Specifically, the active C5 convertase formed on the crystal surface has been demonstrated to trigger the cleavage of C5 protein into C5a and C5b subunits (Russell et al., 1982). C5a exerts potent chemotactic effects on neutrophils and monocytes (Huang et al., 2015). Additionally, it promotes the production of inflammatory cytokines, including IL-1β, and concurrently induces the release of chemokines by endothelial cells and phagocytes (such as macrophages and dendritic cells) (Laudes et al., 2002; Monsinjon et al., 2003).

Cathepsin S (CTSS), a distinctive lysosomal protease, occupies a unique niche within the cysteine cathepsin family. It is intricately involved in diverse pathological processes, including cancer, cardiovascular diseases, and arthritis (Wilkinson et al., 2015). Immune cells, such as macrophages, microglia, B lymphocytes, and dendritic cells, are the sources of CTSS. Notably, under the influence of inflammatory stimuli, these cells can further upregulate the production of CTSS (Petanceska et al., 1996; Zavasnik-Bergant et al., 2001). CTSS plays a crucial role in the immune response. It is essential for antigen processing and presentation, as it can degrade antigenic peptides and antimicrobial peptides, thus being indispensable for the proper functioning of immune cells (Dickinson, 2002). In the extracellular milieu, CTSS exhibits remarkable stability and activity. Even under weakly alkaline pH conditions, it remains enzymatically active. Acting as an elastase, CTSS targets extracellular matrix proteins, such as collagen, and bacterial outer membrane proteins, disrupting their structures and functions (Pietschmann et al., 2013). Proteomic analysis has revealed a crucial finding: the concentration of CTSS in the synovial fluid of gouty arthritis patients is significantly elevated (Fu et al., 2023). This phenomenon suggests that CTSS likely plays a key role in the pathogenesis and disease progression of gouty arthritis.

Recent studies have revealed a significant association between CTSS and complement activation. Specifically, under hyperglycemic conditions, knocking down CTSS can effectively inhibit the expression of C3 and C5 in human umbilical vein endothelial cells and suppress the activation of C3a and C5a, thus alleviating endothelial inflammation (Sayed et al., 2023). It is further hypothesized that CTSS may affect the level of C5a by regulating complement activation, thereby influencing the progression of gouty arthritis.

Our research group has long been dedicated to the study of bioactive molecules with potential anti-inflammatory properties in parasites. In prior investigations, we screened a series of proteins from the human intestinal nematode, *Ancylostoma duodenale*. *Ancylostoma duodenale* cysteine protease inhibitor 2 (CPI2) was identified as a promising candidate, as it demonstrated significant inhibitory effects on CTSS in *in-vitro* assays (Sayed et al., 2023) and also showed a reduction in C5a levels in preliminary *in-vivo* experiments (the data haven't been published yet). Building on these findings, we hypothesized that CPI2 might be efficacious in treating gouty arthritis. In this study, we investigated the protective effect of CPI2 against MSU-induced gouty arthritis using wild-type (WT) and C5aR1 knockout (C5aR1^−/−^) mice and preliminarily evaluated the underlying mechanisms.

## 2 Materials and methods

### 2.1 Recombinant proteins expression

Our research group had previously constructed an *Escherichia coli* (*E. coli*) strain capable of expressing the CPI2 recombinant protein, which was stored at −80°C (Shao et al., 2022). The constructed *E. coli* strains were induced to express. The primer sequences of CPI2 are detailed in Table 1. The strains were inoculated into LB liquid medium containing ampicillin (100 μg/mL, TaKaRa Biotechnology (Dalian) Co., Dalian, China) and cultured at 37°C with a shaking speed of 150 rpm until the optical density (OD) reached approximately 0.6. Subsequently, isopropyl β-D-1-thiogalactopyranoside (IPTG, 100 μg/mL, TaKaRa Biotechnology (Dalian) Co., Dalian, China) was added, and the expression was induced at 35°C for 6 h. After induction, the bacteria were collected by centrifugation at 6,000 × g for 30 min. The bacterial pellet was resuspended in LEW buffer (17.9 g Na_2_HPO_4_•12H_2_O, 17.5 g NaCl, dissolve them in 800 mL of double-distilled water, adjust the pH value to 7.0 with NaOH, and then add double-distilled water to make the volume up to 1 L), and ultrasonic disruption was performed at 30 Hz for 16 min (with a cycle of 5 s of sonication and 3 s of pause). Finally, the lysate was centrifuged at 12,000 × g for 30 min, and the supernatant containing the total protein solution was collected for subsequent experiments.

**TABLE 1:** Amplification primers for the expression of mature CPI2.

Protein	Primer	Sequence (5′-3′)
CPI2	CPI2-1e	CCG​AAT​TCC​AAG​TGA​TGA​CTG​GAG
	CPI2-2e	GCA​AGC​TTC​TCA​AGC​AGA​AAC​GTC

### 2.2 Protein purification

The total protein solution was mixed thoroughly with Ni - NTA resin (QIAGEN, Germany) at a ratio of 20:1 (v/v). Subsequently, the unbound impurities were washed with 10–20 column volumes of Wash Buffer (50 mM PBS, 0.3 M NaCl, 30 mM imidazole). The protein was eluted until the conductivity was equilibrated. The fusion protein was then digested with SUMO protease at a molar ratio of 150:1. Finally, the target protein was eluted with 5–10 column volumes of LEW buffer. The CPI2 obtained by Ni-NTA affinity chromatography was further purified by SP Bio-sep FF ion exchange chromatography (Xi’an Baosai Hengcheng Bioengineering Co., Ltd., China).

### 2.3 The inhibitory activity of CPI2 against CTSS

The experimental methods used to evaluate the inhibitory activity of CPI2 against CTSS have been described in detail in our previously published study (Sayed et al., 2023). In short, a 100 μL reaction system was established. It consisted of 10 μL of either Buffer or CPI2, 50 μL of human CTSS (Calbiochem, Germany) that had been pre-incubated at 37°C for 15 min to reach a final concentration of 0.5 nmol/L, and 40 μL of a fluorescent substrate which was also incubated at 37°C, with a concentration of 4 μmol/L. The substrate employed for the detection of CTSS protease activity was Mca - Gly - Arg - Trp - Pro - Pro - Met - Gly - Leu - Pro - Trp - Glu - Lys (Dnp) - D - Arg - NH_2_ (Calbiochem, Germany). The excitation wavelength (λ_ex_) was set at 340 nm, and the emission wavelength (λ_em_) was set at 405 nm. The pH of the buffer in the reaction system was carefully adjusted to 6.5 to optimize the reaction conditions. Regarding the operation procedure, once the substrate was added to the reaction system and thoroughly mixed, the emission wavelength (λ_em_) was immediately measured using a Cytation 5 multi-function microplate reader (BioTek, United States). After a 5 min interval, the emission wavelength (λ_em_) was measured again. The disparity between these two λ_em_ values was regarded as the reaction rate over the 5 min period. The reaction rate of the control group was denoted as V_0_, while the reaction rate after the addition of CPI2 was labeled as V. To ensure the reliability of the data, each reaction rate was determined in triplicate wells. The inhibitory impact of CPI2 on the activity of CTSS at various concentrations was systematically examined. Prism v.8.0 (GraphPad Software, San Diego, California, United States) software was then utilized for comprehensive data analysis, precise curve fitting, accurate plotting, and ultimately, the determination of the half-maximal inhibitory concentration (*IC*_*50*_) value representing the inhibitory effect of CPI2 on CTSS.

### 2.4 Preparation of MSU crystals

The MSU crystals were prepared under pyrogen-free conditions according to a previously reported protocol (Fang et al., 2021). Briefly, 25 g of uric acid (Sigma - Aldrich, United States) was dissolved in 200 mL of boiling water by adding 6.0 mL of 1 mol/L NaOH. Subsequently, the pH of the resulting solution was carefully adjusted to 7.2 using hydrochloric acid (HCl). After that, the solution was gently stirred at room temperature to facilitate cooling and then stored at 4°C overnight to allow for crystal precipitation. The following day, the precipitate was separated from the solution through filtration and then dried under mild heat conditions. Finally, the obtained MSU crystals were resuspended in phosphate - buffered saline (PBS) to achieve a concentration of 50 mg/mL. This meticulous preparation process ensures the high - quality and reproducibility of the MSU crystals for subsequent experimental use.

### 2.5 Animals

Male C57BL/6 wild-type (WT) and C5aR1 knockout (C5aR1^−/−^) mice on a C57BL/6 genetic background were purchased from Liaoning Changsheng Biotechnology Co., Ltd. (Liaoning, China) and Shanghai Model Organisms Center, Inc. (Shanghai, China), respectively. Mice were maintained in a controlled environment (temperature: 22°C ± 1°C; humidity: 55% ± 5%) on a 12 h light-dark cycle with *ad libitum* access to food and water. All experimental operations with mice followed the National Guidelines for Experimental Animal Care and Use in China. The associated animal protocols were approved by the Laboratory Animal Ethics Committee of Guangdong Medical University (Guangdong, China) (Approval number: GDMU-2023-000061). After 7 days of acclimation, sixty wild-type (WT) mice were randomly divided into six groups using the random number method (10 mice in each group): (i) Sham; (ii) MSU (50 mg/mL, 40 μL); (iii) Colchicine (Col, 0.5 mg/kg, orally gavage after MSU treatment); (iv) CPI2 Low-dose (CPI2-L, 0.5 mg/kg, intravenous injection after MSU treatment); (v) CPI2 Middle-dose (CPI2-M, 1 mg/kg, intravenous injection after MSU treatment) and (ⅵ) CPI2 High-dose (CPI2-H, 2 mg/kg, intravenous injection after MSU treatment). Eighteen mice (C5aR1^−/−^) were randomly divided into 3 groups (6 mice in each group): (ⅶ) Sham; (ⅷ) MSU (50 mg/mL, 40 μL) and (ⅸ) CPI2 Middle-dose (CPI2-M, 1 mg/kg, intravenous injection after MSU treatment). The endotoxin content of MSU and CPI2, detected by the Limulus amebocyte lysate reagent (Zhanjiang A & C Biological Ltd., China), is less than 5 EU/mL.

### 2.6 Animal model of acute gouty arthritis with MSU crystals in mice

Mice were anesthetized by inhalation of 5% isoflurane. Subsequently, MSU crystals (2 mg in 40 μL of normal saline) were injected into the right footpad of each mouse. Sham mice were injected with the same volume of sterile saline into the right foot pad. After treatment with MSU for 24 h, the thickness of the foot was measured by two researchers who were unaware of the grouping situation using an electronic caliper. The mice had their blood collected from the retroorbital venous plexus by inhaling isoflurane, and then were euthanized by cervical dislocation. The foot pad tissues were collected. The parameters of inflammation and oxidative stress were detected 24 h after the injection of MSU crystals. Investigators were blinded to the group allocation when assessing the results of animal studies.

### 2.7 Hematoxylin-eosin (H&E) staining

For histological evaluation, sagittal sections of the footpads were fixed in 10% neutral-buffered formalin for 48 h. The tissues were embedded in paraffin after decalcification with 0.5 M EDTA and then sectioned. Finally, the tissues were subjected to H&E staining (Wang et al., 2019).

### 2.8 Enzyme-linked immunosorbent assay (ELISA)

The serum levels of cytokines (TNF-α, IL-1β, IL-6, and IL-10), C5a, and CTSS were measured using ELISA kits (R&D Systems, United States; Signalway Antibody, United States). The concentrations of TNF-α, IL-1β, IL-6, IL-10, C5a, and CTSS were determined according to the manufacturer’s instructions. The OD value of each well was determined at 450 nm.

### 2.9 Detection of glutathione peroxidase (GSH-Px), superoxide dismutase (SOD) and malondialdehyde (MDA) levels in serum

The activities of glutathione peroxidase (GSH-Px) and superoxide dismutase (SOD) and the level of malondialdehyde (MDA) in serum were determined accurately. These measurements were performed using the relevant assay kits from Nanjing Jiancheng Bioengineering Institute Co., Ltd. (Nanjing, China), following the manufacturer’s detailed instructions precisely. Specifically, for the detection of GSH-Px activity, the absorbance (OD) value of each well was measured at 412 nm; for SOD activity, the OD value was determined at 450 nm; and for the MDA level, the OD value was assessed at 532 nm, respectively.

### 2.10 Immunohistochemical analysis

Immunohistochemical staining for C5aR1 protein was performed. Paraffin-fixed sections were subjected to antigen retrieval by boiling in 1% citrate buffer (pH 6.0) for 10 min and then incubated in 3% hydrogen peroxide for 10 min at room temperature to block endogenous peroxidase activity. Subsequently, the sections were incubated in a protein-free blocking buffer at room temperature for 20 min to minimize background non-specific staining. The expression of C5aR1 (1:50, Signalway Antibody, United States) was detected according to the instructions of the immunohistochemistry kit (Beijing Solarbio Science & Technology Co., Ltd., China). Two researchers who were unaware of the grouping situation took photos of the pathological sections and calculated the Mean Optical Density (MOD) using Image-Pro plus 6.0 software (IPP) to analyze the semi-quantitative expression of C5aR1. The measurement steps are as follows: Open the IPP, import the image for analysis, calibrate the optical density value, select the measurement area, and obtain the MOD. Measure the MOD of each sample 3 times repeatedly and calculate the average value.

### 2.11 Statistical analysis

All data were expressed as the mean ± SD. Prism v.8.0 (GraphPad Software, San Diego, California, United States) software was used for the analysis. One-way analysis of variance was used for multiple group analysis. The two-tailed unpaired *t*-test was used to analyze the data from two groups. *P* values of less than 0.05 and 0.01 were considered significant.

## 3 Results

### 3.1 Generation of the recombinant protein

**FIGURE 1:**
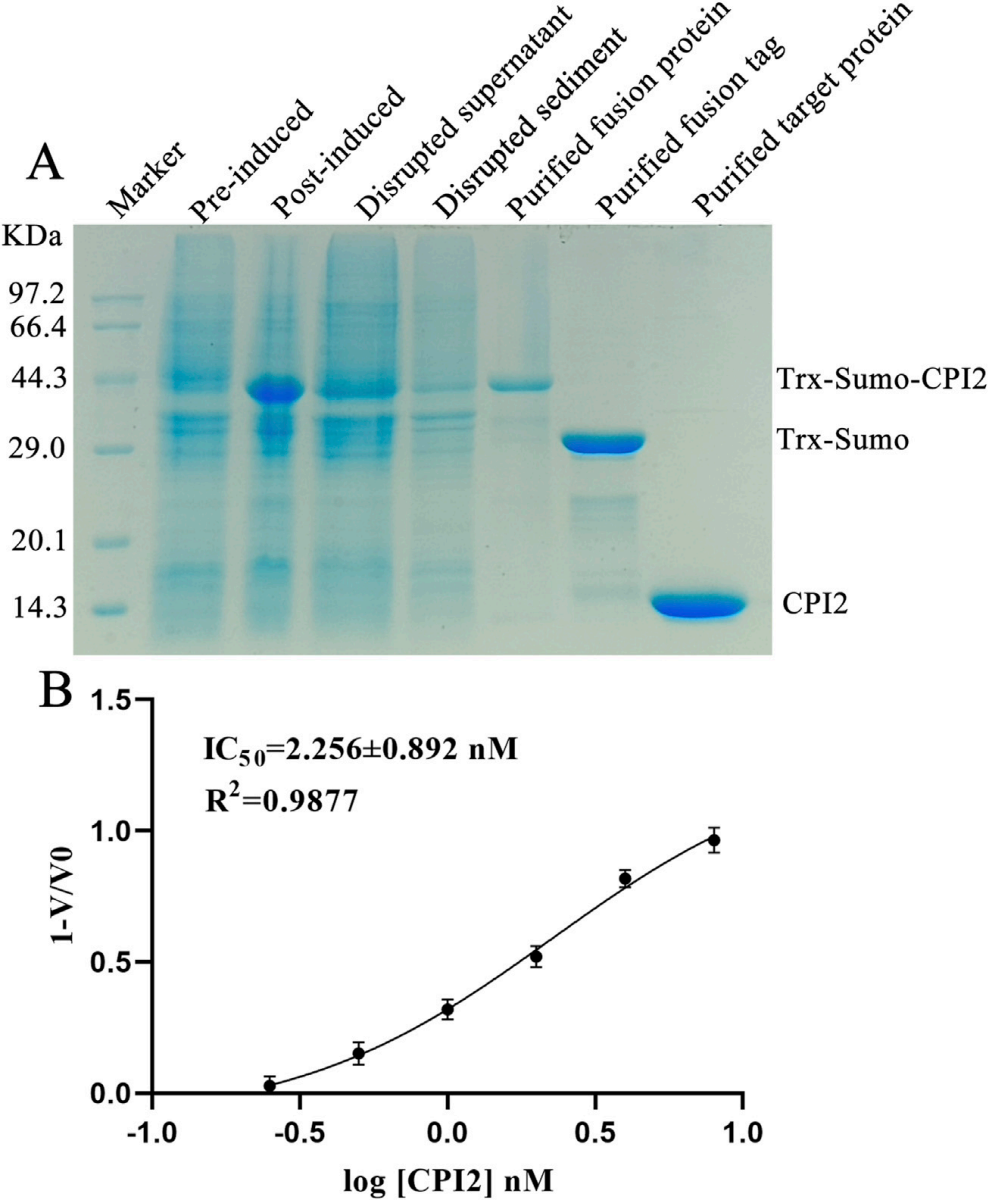
Purified CPI2 and Its IC_50_ against Cathepsin S. **(A)** Purification analysis of CPI2 by SDS-PAGE electrophoresis; **(B)** Dose-response relationship between log[CPI2] (nM) and the response value 1-V/V_0_, with the IC_50_ of CPI2 against Cathepsin S presented; Data are expressed as the mean ± SEM (n = 3).

To obtain the recombinant CPI2 protein for subsequent experiments, we carried out the protein expression and purification process. The purified CPI2 protein migrated as a distinct band at approximately 14 kDa in SDS-PAGE, as shown in Figure 1A. The purity of the purified CPI2 protein was determined to be > 98%. Using the BCA kit (Sangon Biotech Co., Ltd., Shanghai, China), the protein concentration was measured as 2.0 mg/mL.

### 3.2 CPI2 inhibits the activity of CTSS

To assess the inhibitory effect of CPI2 on CTSS, enzyme activity assays were conducted. Enzyme activity assays demonstrated that the inhibitory effect of CPI2 on human CTSS was dose-dependent, indicating a potent inhibitory capacity of CPI2 on CTSS. The half-maximal inhibitory concentration (*IC*_*50*_) was calculated to be 2.256 nM, as depicted in Figure 1B.

### 3.3 CPI2 alleviates footpad swelling induced by MSU

**FIGURE 2:**
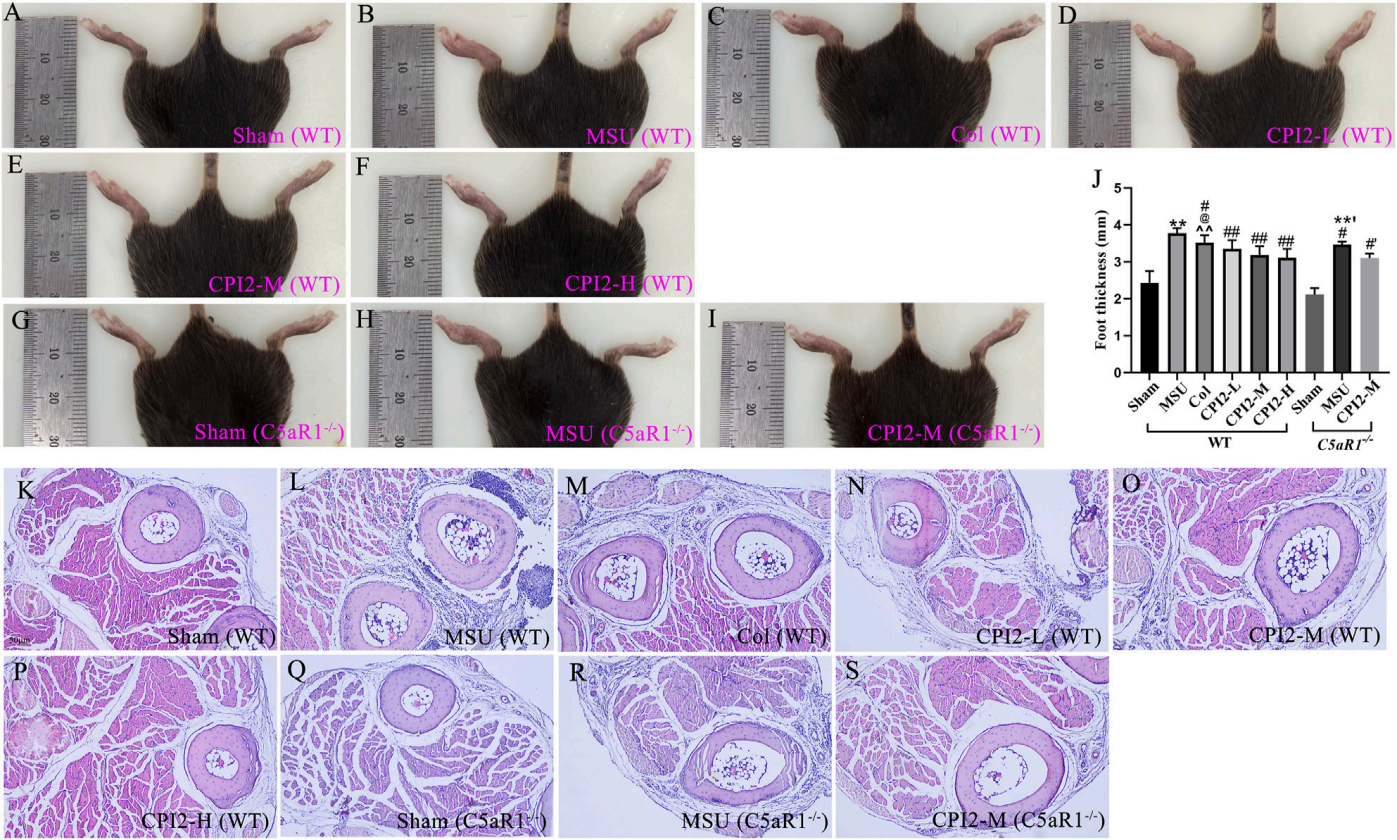
CPI2 alleviates the swelling of the ankle and footpad in MSU-induced acute gouty arthritis. **(A–I)** Representative images of the hind paw ankle and footpad of WT and C5aR1−/− mice, captured 24 h after intraplantar injection of MSU; **(J)** Hind paw thickness measured by caliper in each group of mice at 24 h after MSU injection; Data are expressed as the mean ± SEM (n ≥ 6); **P < 0.01 versus the sham group (WT); #*P* < 0.05, ##*P* < 0.01 versus the MSU group (WT); @*P* < 0.05 versus the CPI2-M group (WT); ^^*P* < 0.01 versus the CPI2-H group (WT); **’*P* < 0.01 versus the sham group (C5aR1−/−); #’*P* < 0.05 versus the MSU group (C5aR1−/−); **(K–S)** Representative HE-stained images of hind paw footpads from WT and C5aR1−/− mice 24 h after intraplantar MSU injection.

To evaluate the effect of CPI2 on MSU-induced footpad swelling, we measured the thickness of the right feet of mice in the sham group and the treated group. As shown in Figures 2A–I, 24 h after the injection of MSU crystals, significant redness, edema (*P* < 0.01) and deformity were observed in the right ankle joints and paws of the MSU group mice compared with those of the sham group mice. Moreover, as shown in Figure 2J, this swelling was reduced after treatment with CPI2 (0.5–2 mg/kg) and Col (0.5 mg/kg) (*P* < 0.05). Histological analysis of the footpad tissues revealed a large number of inflammatory cell infiltrations in the MSU-treated group (Figures 2L,R) compared with the sham group (Figures 2K, Q). Treatment with CPI2 and Col visibly suppressed the influx of inflammatory cells (Figures 2M–P,S). Furthermore, we explored the effect of C5aR1 on MSU-induced gouty arthritis and verified whether CPI2 alleviates gouty arthritis through C5aR1. Compared with the MSU group (WT), the mice in the MSU group (C5aR1^−/−^) showed significantly ameliorated symptoms of redness, swelling (*P* < 0.05), and deformity in the right ankle joints and paws, along with a reduced influx of inflammatory cells (Figures 2B,H,J,L,R). These results suggest that MSU crystal-induced ankle joints and paw swelling was suppressed by CPI2. C5aR1 is involved in the progression of acute gouty arthritis induced by MSU.

### 3.4 CPI2 reduces the levels of inflammatory cytokines

**FIGURE 3:**
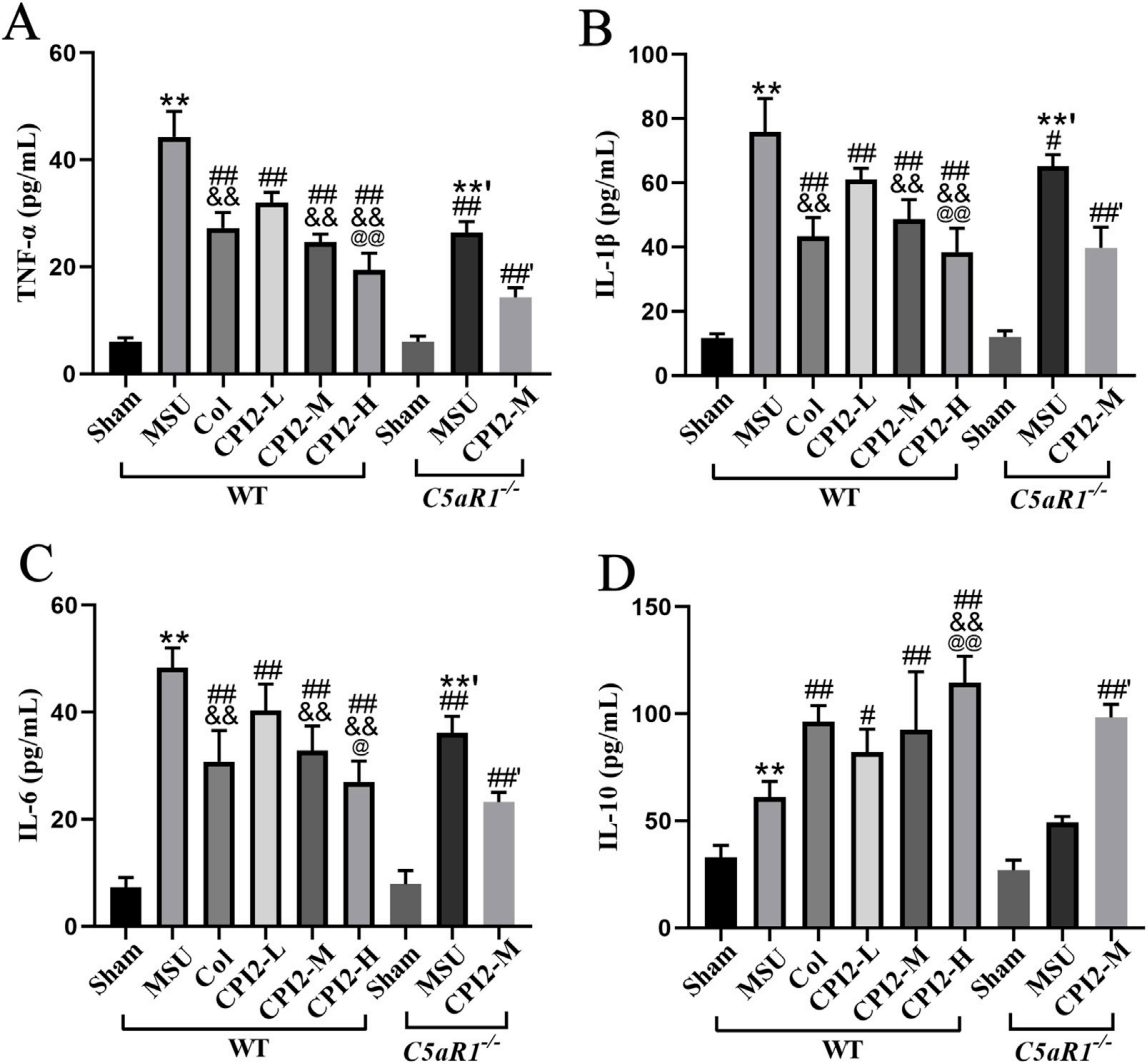
CPI2 ameliorates inflammatory cytokines in serum. **(A–D)** The levels of TNF-α, IL-1β, IL-6, and IL-10 in WT and C5aR1^−/−^ mice measured by ELISA, respectively; Data are expressed as the mean ± SEM (n ≥ 6); **P < 0.01 versus the sham group (WT); ^#^ *P* < 0.05, ^##^ *P* < 0.01 versus the MSU group (WT); ^&&^ *P* < 0.01 versus the CPI2-L group (WT); ^@^ *P* < 0.05, ^@@^ *P* < 0.01 versus the CPI2-M group (WT); **’*P* < 0.01 versus the sham group (C5aR1^−/−^); ^##’^ *P* < 0.01 versus the MSU group (C5aR1^−/−^).

To investigate the anti-inflammatory effect of CPI2, we measured the serum levels of cytokines TNF-α, IL-1β, IL-6, and IL-10. After the injection of MSU for 24 h, the levels of these cytokines were detected by ELISA. The ELISA results (Figures 3A–D) demonstrated that MSU induced a significant elevation in TNF-α, IL-1β, IL-6, and IL-10 levels (*P* < 0.01). However, treatment with CPI2 in a dose-dependent manner markedly suppressed the production of pro-inflammatory cytokines (TNF-α, IL-β, and IL-6) (*P* < 0.01) while enhancing the production of the anti-inflammatory cytokine IL-10 (*P* < 0.05). In comparison with the MSU group, Col significantly reduced the levels of pro-inflammatory cytokines (TNF-α, IL-β, and IL-6) (*P* < 0.01). Simultaneously, it increased the production of the anti-inflammatory cytokine IL-10 (*P* < 0.01). Compared with the MSU group (WT), the levels of pro-inflammatory factors (TNF-α, IL-1β, and IL-6) in the MSU group (C5aR1^−/−^) were significantly reduced (*P* < 0.05). The findings unequivocally demonstrated that treatment with CPI2 effectively curbed the production of the key pro-inflammatory cytokines, namely, TNF-α, IL-1β, and IL-6, while concurrently fostering the synthesis of the anti-inflammatory cytokine IL-10. These cytokines play pivotal roles in both the onset and advancement of acute gouty arthritis. C5aR1 is involved in the inflammatory progression of acute gouty arthritis induced by MSU. Knocking out or blocking C5aR1 helps alleviate the inflammation of acute gouty arthritis.

### 3.5 CPI2 alleviates oxidative stress injury

**FIGURE 4:**
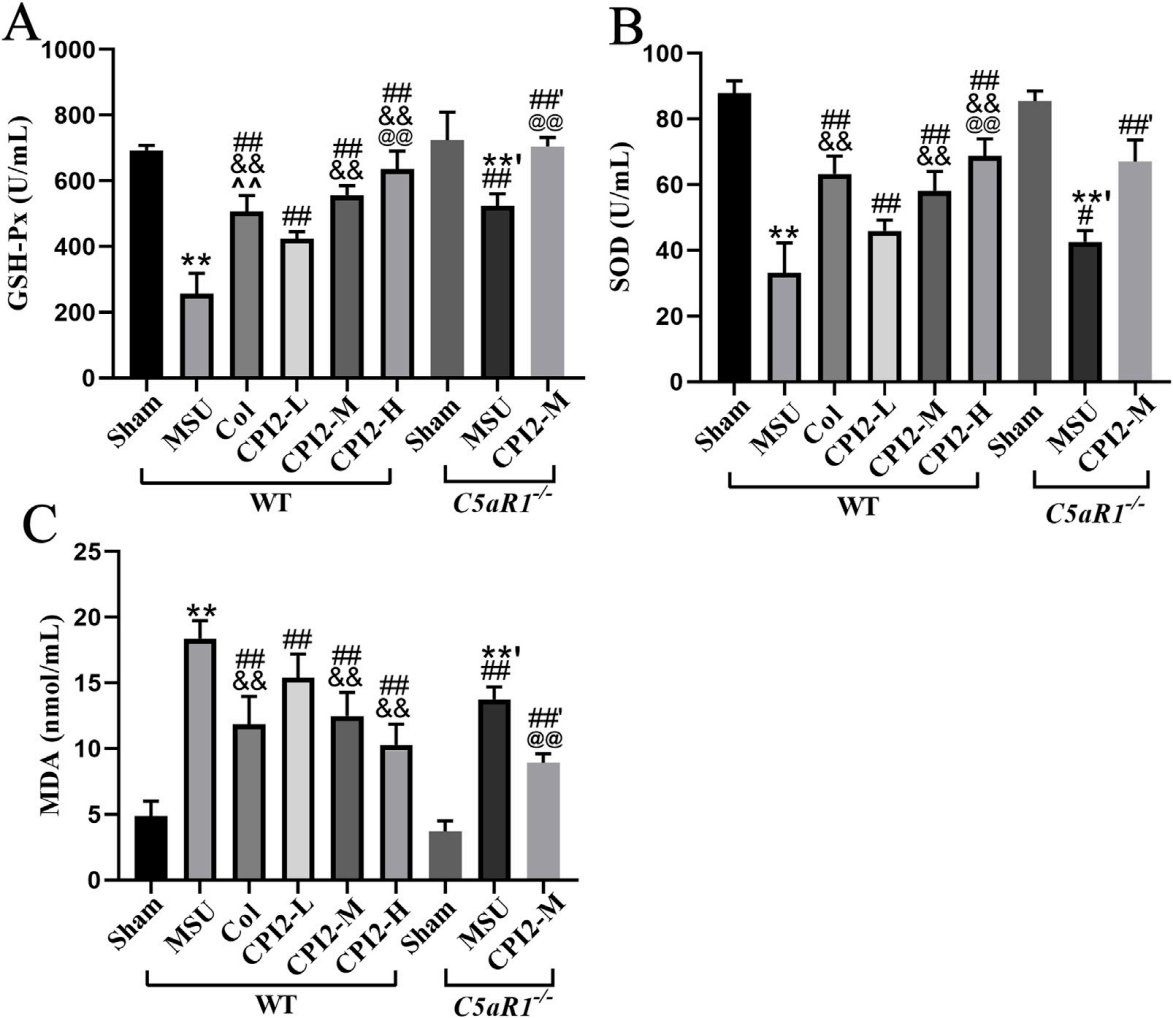
CPI2 alleviates oxidative stress in serum. The activity of GSH-Px **(A)** and SOD **(B)**, and the content of MDA **(C)** in WT and C5aR1^−/−^ mice at 24 h after MSU injection, measured by chemiluminescence assay; Data are expressed as the mean ± SEM (n ≥ 6); **P < 0.01 versus the sham group (WT); ^#^ *P* < 0.05, ^##^ *P* < 0.01 versus the MSU group (WT); ^&&^ *P* < 0.01 versus the CPI2-L group (WT); ^@@^ *P* < 0.01 versus the CPI2-M group (WT); **’*P* < 0.01 versus the sham group (C5aR1^−/−^); ^##’^ *P* < 0.01 versus the MSU group (C5aR1^−/−^).

To explore the effect of CPI2 on oxidative stress in acute gouty arthritis, we measured several oxidative stress - related indicators in mice. As shown in Figures 4A–C, 24 h after the injection of MSU, the activities of GSH-Px and SOD in serum decreased significantly (*P* < 0.01), while the content of MDA increased remarkably (*P* < 0.01). However, after treatment with CPI2, the activities of these enzymes in serum increased in a dose-dependent manner (*P* < 0.01), and the MDA content decreased in a dose-dependent manner (*P* < 0.01).

Compared with the MSU group (WT), the MSU group (C5aR1^−/−^) exhibited a significant increase in the activities of GSH-Px and SOD (*P* < 0.05), along with a substantial decrease in the content of MDA (*P* < 0.01). Similarly, when compared to the CPI2-M group (WT), the CPI2-M group (C5aR1^−/−^) also showed a marked rise in the activities of GSH-Px (*P* < 0.01), and a significant reduction in the MDA content (*P* < 0.01). These results suggest that CPI2 can ameliorate oxidative stress, demonstrating its antioxidant properties. C5aR1 is involved in the oxidative stress process of acute gouty arthritis induced by MSU.

### 3.6 CPI2 reduces the levels of C5a and CTSS

**FIGURE 5:**
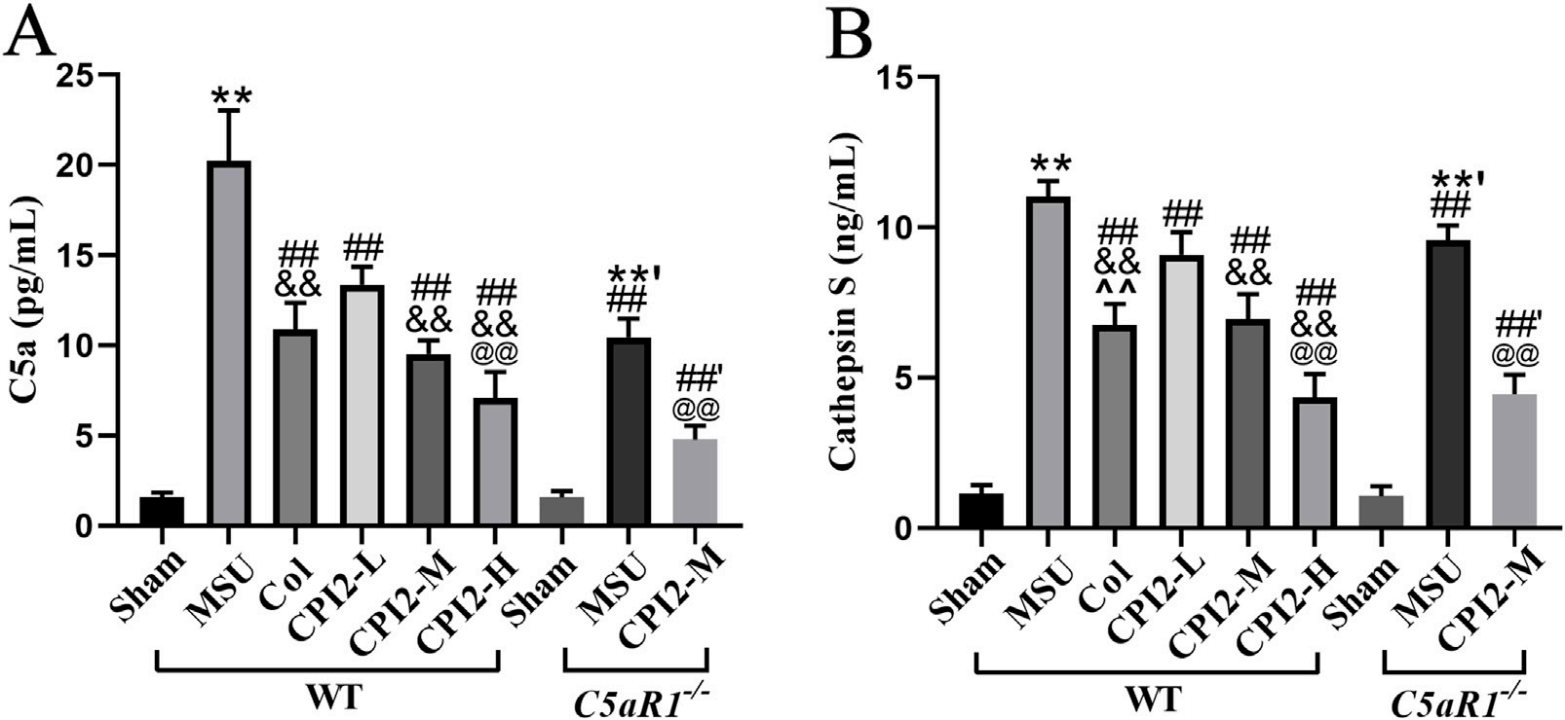
CPI2 reduces the levels of C5a and cathepsin S in serum. **(A, B)** The levels of C5a and cathepsin S in WT and C5aR1^−/−^ mice measured by ELISA, respectively; Data are expressed as the mean ± SEM (n ≥ 6). **P < 0.01 versus the sham group (WT); ^#^ *P* < 0.05, ^##^ *P* < 0.01 versus the MSU group (WT); ^&&^ *P* < 0.01 versus the CPI2-L group (WT); ^@^ *P* < 0.05, ^@@^ *P* < 0.01 versus the CPI2-M group (WT); **’*P* < 0.01 versus the sham group (C5aR1^−/−^); ^##’^ *P* < 0.01 versus the MSU group (C5aR1^−/−^).

To further explore the effect of CPI2 on the complement system and CTSS release, we used the ELISA method to measure the serum levels of C5a and CTSS. Our findings indicated that CPI2 reduced the levels of C5a and CTSS in a dose-dependent manner in mice with acute gouty arthritis induced by MSU (Figures 5A,B) (*P* < 0.01). When compared with the MSU group (WT), the levels of C5a and CTSS in the MSU group (C5aR1^−/−^) were significantly lower (Figures 5A,B) (*P* < 0.01). Similarly, the levels of C5a and CTSS in the CPI2-M group (C5aR1^−/−^) were notably decreased compared with those in the CPI2-M group (WT) (Figures 5A,B) (*P* < 0.01). These results suggest that CPI2 can regulate the complement system and reduce the release of CTSS from inflammatory cells, demonstrating its immunomodulatory and anti-inflammatory properties. C5aR1 is involved in the immunoregulation and inflammation of acute gouty arthritis induced by MSU. CPI2 may alleviate gouty arthritis by reducing the level of CTSS and regulating the C5a-C5aR1 axis.

### 3.7 CPI2 reduces the expression level of C5aR1 protein in paw tissues

**FIGURE 6:**
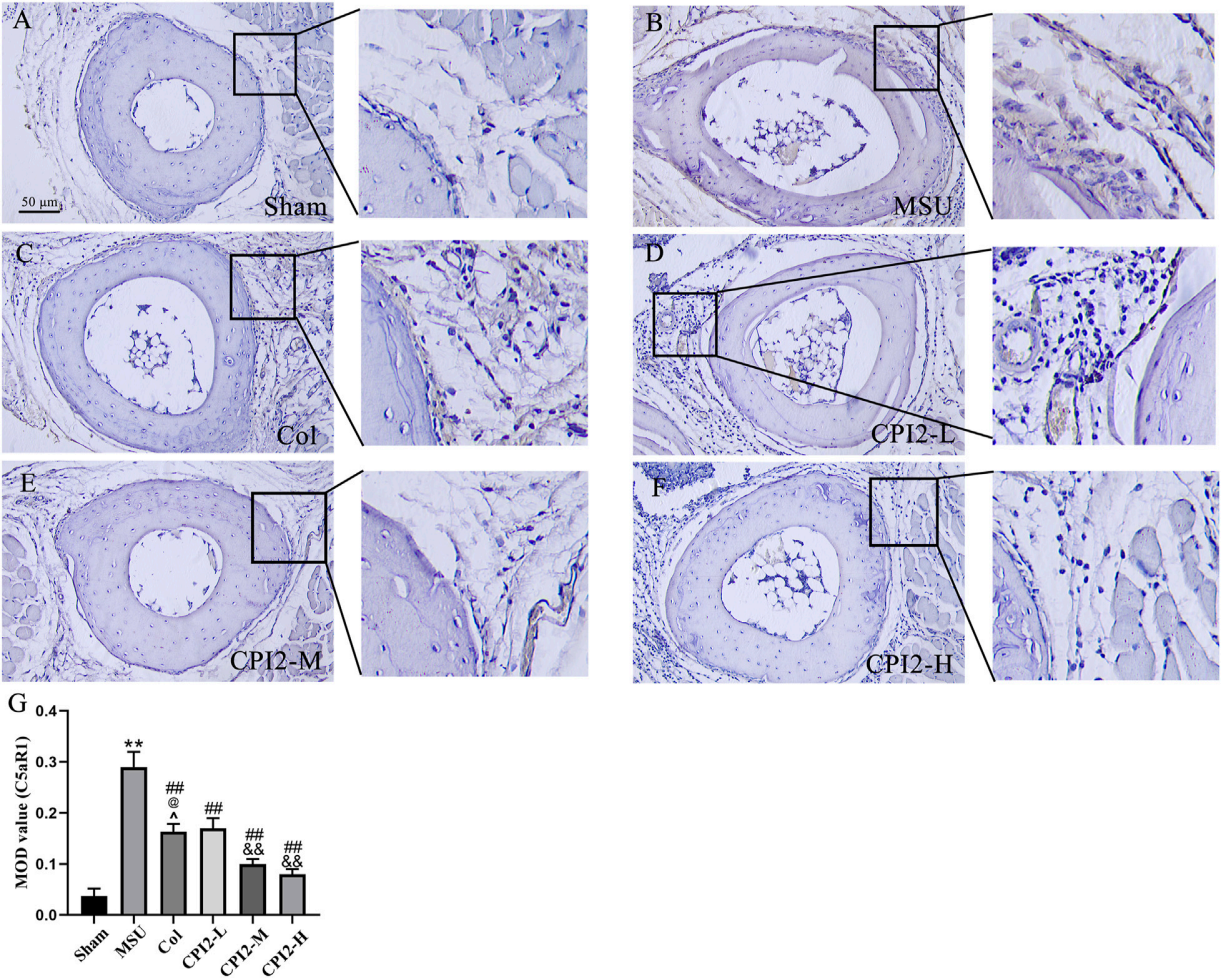
Protein expression of C5aR1 in the footpads. Representative immunohistochemical staining images of C5aR1 in hind paw footpads from the Sham group **(A)**, MSU group **(B)**, Col group **(C)**, CPI2-L group **(D)**, CPI2-M group **(E)**, CPI2-H group **(F)**, respectively. **(G)** Quantitative immunohistochemical results for C5aR1 measured as mean optical density (MOD) values; Data are expressed as the mean ± SEM (n = 3). **P < 0.01 versus the sham group; ^##^ *P* < 0.01 versus the MSU group; ^&&^ *P* < 0.01 versus the CPI2-L group; ^@^ *P* < 0.05 versus the CPI2-M group.

To explore the effect of CPI2 on the expression of C5aR1 protein in paw tissues, immunohistochemistry was performed (Figures 6A,G). The results indicated that CPI2 downregulated the expression level of C5aR1 protein in the paw tissues of mice with MSU-induced acute gouty arthritis in a dose-dependent manner (*P* < 0.01) (Figures 6D,G). These research findings indicate that CPI2 can ameliorate MSU-induced gouty arthritis by regulating the C5a-C5aR1 axis.

## 4 Discussion

Gouty arthritis, an inflammatory disease, is triggered by the deposition of MSU crystals in joints. Numerous studies have confirmed the crucial role of MSU deposition in joints during the pathogenesis of gouty arthritis (Smyth et al., 2022). To explore the effect of CPI2 on acute gouty arthritis, we induced mice with MSU to establish an acute gouty arthritis model. During the development of gouty arthritis, in addition to obvious joint swelling, redness, and pain, during the development of the inflammatory response and gout attacks, MSU crystals can prompt a large number of white blood cells to infiltrate into the joint cavity (Brix et al., 2008). This early pathological feature is specifically manifested as neutrophils rushing into the joint fluid first, followed by the successive entry of monocytes and macrophages. These cells will phagocytize MSU crystals, thereby triggering cell membrane lysis and inflammasome activation (Fan et al., 2021; So and Martinon, 2017), generating oxygen-derived free radicals, and releasing lysosomal enzymes and pro-inflammatory cytokines, mainly including TNF-α, IL-1β, and IL-6 (Martinon and Glimcher, 2006; Martin et al., 2009). Inflammation and oxidative stress assume pivotal roles in the development of gouty arthritis, existing in an intricate and intertwined relationship, collaborating synergistically to propel the advancement of gouty arthritis (Di Giovine et al., 1987). We observed that 24 h after the injection of MSU crystals, the right ankle joints and paws of the mice exhibited significant redness, edema, and deformity. Meanwhile, a large number of inflammatory cells infiltrated, and the levels of pro-inflammatory cytokines (such as IL-1β, IL-6, and TNF-α) increased sharply. Interestingly, we found that the level of IL-10 also increased significantly. IL-10 is an important anti-inflammatory cytokine. The increase in IL-10 induced by MSU may be a self - protective mechanism of the body. However, the specific molecular mechanisms underlying the increase in IL-10 induced by MSU and its comprehensive effects in the complex *in vivo* environment still need to be further investigated in depth. In addition, the activities of SOD and GSH-Px were markedly reduced, while the level of MDA increased significantly. These symptoms are highly consistent with those of murine gouty arthritis described in other related studies (Schweyer et al., 2000; Du et al., 2023), strongly indicating that the murine gouty arthritis model has been successfully established by MSU induction. Moreover, we also found that the injection of MSU could increase the level of CTSS.

Among the lysosomal cysteine cathepsin family, CTSS has unique characteristics. CTSS has two key features differentiating it from other cysteine cathepsins. Firstly, its expression is relatively restricted, mainly in immune cells like professional antigen - presenting cells, B cells, dendritic cells, and macrophages. Secondly, it can maintain catalytic activity in a neutral to weakly alkaline pH environment (Cheng et al., 2022). CTSS exhibits good enzymatic activity within a relatively wide pH range (pH 4.5 - 8) (Chen et al., 2019). Lysosomal cysteine cathepsins are closely related to gouty arthritis. Cathepsin B and cystatin C play a pro-inflammatory role in gouty arthritis of the knee joint (Chu et al., 2010). With the continuous deepening of research, more and more evidence shows that abnormal expression of CTSS exists in a variety of situations and disease states. This discovery has made CTSS a biomarker and therapeutic target with great potential. It is worth noting that some studies have clearly pointed out that the level of CTSS in gouty arthritis patients is significantly elevated (Fu et al., 2023), which may open up new directions for the diagnosis and treatment of gout. We successfully obtained a CTSS inhibitor, CPI2, from *A. duodenale* using gene recombination technology and protein purification technology. Its purity can be > 98%, and it has good inhibitory activity against CTSS (*IC*_*50*_ = 2.256 nM). During the preliminary pre-experiment stage, we set up a series of concentration gradients for preliminary exploration and found that the three concentrations of CPI2 (0.5, 1.0, and 2.0 mg/kg) can effectively reduce the symptoms of acute gouty arthritis, and the effect is dose-dependent. Therefore, we determined to use the above three concentrations of CPI2 to conduct this experiment. We found that CPI2 can improve the symptoms and indicators of the mouse model of acute gouty arthritis induced by MUS in a dose-dependent manner. These findings suggest that CPI2 may alleviate inflammation and oxidative stress by inhibiting the activity of CTSS, thereby contributing to the improvement of gouty arthritis.

In addition to its impact on CTSS, we also investigated the effect of CPI2 on the C5a-C5aR1 axis, which is closely related to the inflammatory process of gouty arthritis. Recent studies have pointed out that targeting C5a may be of great benefit for the treatment of acute gouty arthritis (Khameneh et al., 2017). MSU crystals can not only trigger gouty arthritis but also activate the complement system, thus regulating the inflammatory response (Doherty et al., 1983). In in vitro experiments, C5a can significantly enhance the ability of peritoneal macrophages and human monocytic cell lines, which are stimulated by lipopolysaccharide and simultaneously exposed to MSU, to release IL-1β (Khameneh et al., 2017). In the *in-vivo* environment, C5a induced by MSU mediates neutrophil recruitment in mice and promotes IL-1β production at the inflammation site. Significantly, treatment with a C5a receptor antagonist in mice markedly ameliorates the C5a-mediated effects described above (Khameneh et al., 2017). In addition, C5a generated after MSU-induced activation of the complement system promotes the production of a large amount of reactive oxygen species (ROS) and pro-inflammatory cytokines, thus further exacerbating the progression of gouty arthritis (Khameneh et al., 2017). In contemporary research, C5aR1 is regarded as the primary receptor through which C5a exerts its functions (Wang et al., 2023). C5aR1 is extensively expressed on the surfaces of diverse cell types, particularly immune cells such as neutrophils, monocytes, and macrophages (Thurman, 2017). Upon binding to C5aR1, C5a activates a cascade of intracellular signaling pathways, triggering multiple biological responses. During the inflammatory process, this binding event promotes the chemotaxis of neutrophils and monocytes, facilitating their recruitment to the site of inflammation. Concurrently, it stimulates cells to secrete inflammatory cytokines, including IL-1β and IL-6, which further exacerbate the inflammatory response (Monk et al., 2007). Our study reveals that 24 h after MSU injection in mice, the serum C5a level in mice with acute gouty arthritis increases significantly, and concurrently, the expression of C5aR1 protein is upregulated. When C5aR1 is knocked out, the symptoms of mice with acute gouty arthritis are significantly alleviated, and the inflammatory and oxidative stress indexes are obviously improved, which indicates that the C5a - C5aR1 axis is involved in the progression of acute gouty arthritis. Notably, after administration of CPI2, the C5a level in the serum of mice decreases in a dose - dependent manner, and the expression of C5aR1 protein is downregulated. This result suggests that CPI2 may play a role in improving acute gouty arthritis by regulating the C5a-C5aR1 axis. CPI2 can also improve the inflammatory factors and oxidative stress indexes in C5aR1^−/−^ mice induced by MSU, suggesting that CPI2 has other target sites, such as CTSS.

Colchicine, a water-soluble alkaloid, is a commonly recommended drug in clinical practice for the treatment of acute gout and the prevention of acute attacks during the initial stage of uric acid-lowering therapy (Terkeltaub, 2003). Currently, it is widely believed in the academic community that colchicine mainly blocks microtubule formation, effectively inhibiting the migration of neutrophils and other white blood cells triggered by MSU crystals to the inflamed site (Cronstein et al., 1995). This, in turn, exerts a significant anti-inflammatory effect and reduces the inflammatory response during acute gout attacks. However, CPI2 may exert its anti-gout effect by inhibiting the activity of CTSS. During the inflammatory process of gout, MSU crystals stimulate inflammatory cells, leading to an increase in the activity of CTSS. CTSS will further activate the inflammatory signaling pathway, promoting the release of pro-inflammatory cytokines and exacerbating the inflammatory response. CPI2 can specifically inhibit the activity of CTSS, block the excessive activation of the inflammatory signaling pathway, reduce the production of pro-inflammatory cytokines, and may also regulate the C5a-C5aR1 axis, thereby alleviating the inflammatory and oxidative stress responses and improving the symptoms of gouty arthritis. Compared with colchicine, CPI2 is superior in reducing the swelling of the footpad in acute gouty arthritis induced by MSU, increasing the activity of GSH-Px, and decreasing the levels of CTSS and C5aR1. Therefore, CPI2 may have greater potential in the therapeutic effect of acute gout. The adverse reactions of colchicine in the treatment of gouty arthritis are also relatively common, mainly manifested as gastrointestinal reactions, muscle and peripheral nerve disorders, bone marrow suppression, etc (Stamp et al., 2024). Since we haven't conducted the toxicological experiments of CPI2, whether CPI2 has better potential advantages in terms of side effects still requires further investigation.

Overall, our research findings indicate that CPI2 mainly exerts its effects by inhibiting the activity of CTSS and regulating the C5a-C5aR1 axis. This leads to a significant reduction in the levels of pro-inflammatory cytokines (IL-1β, IL-6, and TNF-α) and MDA, while simultaneously elevating the level of the anti-inflammatory cytokine IL-10 and the activity of SOD and GSH-Px. As a result, CPI2 ameliorates the symptoms of MSU-induced acute gouty arthritis. In view of this, CPI2 holds promise as a new candidate drug for the treatment of gouty arthritis, providing novel ideas and solutions for the treatment of this disease.

## 5 Conclusion

Our *in-vivo* experimental model has for the first time confirmed that CPI2 can effectively reduce the swelling induced by MSU crystals in mice. Its anti-inflammatory mechanisms are mainly reflected in the following aspects: On the one hand, CPI2 inhibits the release of pro-inflammatory cytokines such as IL-1β, IL-6, and TNF-α, while promoting the secretion of the anti-inflammatory cytokine IL-10. On the other hand, it decreases the levels of MDA and C5a, enhances the activities of SOD and GSH-Px, inhibits the activity of CTSS, downregulates the expression level of C5aR1, and reduces the infiltration of inflammatory cells. These research results fully demonstrate that CPI2 has potential therapeutic effects on gouty arthritis. It may inhibit the activity of CTSS, regulate the C5a-C5aR1 axis, reduce swelling and inflammatory responses, and thus play an inhibitory role in gouty arthritis.

## Data Availability

The original contributions presented in the study are included in the article/supplementary material, further inquiries can be directed to the corresponding authors.
